# Ulcer pressure prevention and opportunity for innovation during the COVID-19 crisis

**DOI:** 10.6061/clinics/2020/e2292

**Published:** 2020-08-28

**Authors:** Fernando Souza Nani, Kelly Cristina Stéfani, Fabio de Freitas Busnardo, Gustavo Gomes Ribeiro Monteiro, Maria Gabriela Guimarães Ribeiro dos Santos, Vanderley Moacyr John, Douglas Gouvêa, Maria José C. Carmona

**Affiliations:** I Hospital das Clinicas HCFMUSP, Faculdade de Medicina, Universidade de Sao Paulo, Sao Paulo, SP, BR.; II Escola Politecnica, Universidade de Sao Paulo, Sao Paulo, SP, BR.; III Centro de Inovacao da USP (Inova USP), Sao Paulo, SP, BR.

The coronavirus disease (COVID-19) pandemic has put the health system under stress in many places around the world. Worldwide, the shortage of health supplies, including medical devices and medications, has become a reality in daily practice. Restrictions on production related to the pandemic itself has increased the demand for treatment methods/strategies, and the dependence on imports has led to a parallel crisis in the healthcare system (1).

However, crises provide excellent opportunities for innovation in processes, products, and technologies, some of them resulting in long-lasting solutions. In Brazil, Hospital das Clínicas da Faculdade de Medicina da Universidade de São Paulo (HCFMUSP) managed the pandemic by increasing the number of intensive care units to treat severe cases of COVID-19.

The new scenario led to the challenge of pressure ulcer prevention. Pressure ulcers are a global issue associated with a significant health burden, and their prevention should be a priority in all healthcare settings. Along with the regular risk of pressure ulcers in sacral and bony prominences, frequent prone positioning during COVID-19 treatment poses an additional risk (2,3). The prone position is beneficial for specific surgical approaches and is also a treatment strategy for respiratory insufficiency (4,5). However, the use of this position requires attention to intrinsic risks, such as an increase in intra-abdominal pressure, limb compartment syndrome, nerve palsies, cardiovascular compromise, vision loss, venous air embolism, tracheal tube dislodgement, and pressure sores (6). The use of specific cushions can help prevent pressure ulcers on the face, thorax, and bony prominences (7).

Prone position ventilation may be effective in recruiting non-aerated alveolar units, and one or more sessions were indicated for about 20% of patients under mechanical ventilation during the COVID-19 outbreak. However, with this protocol, we faced the problem of shortage of specific cushions for adequate pronation (8). This challenge was also an opportunity for innovation.

In view of this scenario and to arrive at a quick solution, Hospital das Clínicas, Escola Politécnica, and Universidade de São Paulo Innovative Centers entered into a public-private partnership aiming at identifying, testing, and producing cushions in a short period of time. Physicians, engineers, nurses, physiotherapists, and designers came together to develop and approve dedicated cushions, using the experience of each professional. Few weeks of intense work resulted in new products, after adequate usability tests. The precise execution of initial innovative ideas was made possible by personnel who assertively conveyed their demands, provided input on areas of improvement in the tests carried out, and participated in convincing frontline health professionals about the importance of such a project despite being overloaded with work. Simple innovations can solve complicated problems. The unpretentious idea of producing new cushions triggered the processes for developing guidelines for prone positioning, prevention of bedsores, and early identification and treatment of pressure injuries. The Innovative Centers from Instituto Central of HCFMUSP and the University of São Paulo had the opportunity to challenge themselves in coordinating intra- and extra-hospital teams that had never communicated with each other before, with the objective of approving and finalizing a project in record time, considering the peculiarities of severe acute respiratory syndrome coronavirus 2 (SARS-CoV-2) transmissibility.

The final product, aside from its humanitarian purpose during the pandemic, could be a future market product used as a substitute for imported cushions, after specific usability tests following the Brazilian Health Regulatory Agency (ANVISA) guidelines. Despite the COVID-19 crisis, a lesson could be learned by universities, companies, and individuals: when we expand and de-bureaucratize relationships beyond institutional boundaries, we generate a smooth and adequate flow for the quick development of ideas. The resultant solutions can radically affect our daily life problems. This is certainly a great takeaway from this pandemic.

## References

[1] Cyranoski D (2020). Mystery deepens over animal source of coronavirus. Nature.

[2] Sung CS, Park JY (2019). A monitoring sensor-based eHealth image system for pressure ulcer prevention. Multimed Tools Appl.

[3] Girard R, Baboi L, Ayzac L, Richard JC, Guérin C, Proseva trial group (2014). The impact of patient positioning on pressure ulcers in patients with severe ARDS: results from a multicentre randomised controlled trial on prone positioning. Intensive Care Med.

[4] Bloomfield R, Noble DW, Sudlow A (2015). Prone position for acute respiratory failure in adults. Cochrane Database Syst Rev.

[5] Munshi L, Del Sorbo L, Adhikari NKJ, Hodgson CL, Wunsch H, Meade MO (2017). Prone Position for Acute Respiratory Distress Syndrome A Systematic Review and Meta-Analysis. Ann Am Thorac Soc.

[6] Kwee MM, Ho YH, Rozen WM (2015). The prone position during surgery and its complications: a systematic review and evidence-based guidelines. Int Surg.

[7] McInnes E, Bell-Syer SE, Dumville JC, Legood R, Cullum NA (2008). Support surfaces for pressure ulcer prevention. Cochrane Database Syst Rev.

[8] Ghelichkhani P, Esmaeili M (2020). Prone Position in Management of COVID-19 Patients; a Commentary. Arch Acad Emerg Med.

